# How malleable are attentional biases in women with body dissatisfaction? Priming effects and their impact on attention to images of women’s bodies

**DOI:** 10.1177/2043808719837137

**Published:** 2019-06-06

**Authors:** Samantha Withnell, Christopher R. Sears, Kristin M. von Ranson

**Affiliations:** University of Calgary, Canada; University of Calgary, Canada; University of Calgary, Canada

**Keywords:** Attentional biases, body appreciation, body dissatisfaction, body image, body satisfaction, eye-tracking

## Abstract

Understanding attentional biases associated with body dissatisfaction can aid in devising and refining programs to reduce body dissatisfaction. This study compared attention to images of women’s bodies before and after a body satisfaction or body dissatisfaction priming task. Attention was assessed using eye-gaze tracking, by measuring participants’ fixations to images of “thin” models, “fat” models, and images of average women over an 8-s presentation. Women with high (*n* = 65) and low (*n* = 43) levels of trait body dissatisfaction, as measured by the Body Shape Questionnaire, were randomly assigned to a body satisfaction or body dissatisfaction priming task. Results indicated that both priming tasks were effective at modifying participants’ state body satisfaction. Women with high body dissatisfaction exhibited an attentional bias to thin and fat model images prior to the priming procedure, replicating previous findings. Contrary to predictions, body dissatisfaction priming increased attention to body images for women with both high and low body dissatisfaction, whereas body satisfaction priming had no effect on attention for either group. These findings show that women with high and low body dissatisfaction are vulnerable to the effects of body dissatisfaction priming.

Body dissatisfaction is common in women. Approximately 70% of women report experiencing some dissatisfaction with their body shape, weight, or appearance (Fiske, Fallon, Blissmer, & Redding, 2014). Dissatisfaction with one’s body size or shape is linked to substance abuse (Schwinn, Schinke, Hopkins, & Thom, 2016) and poor peer relationships and self-esteem (Davison & McCabe, 2006). Body dissatisfaction has also been identified as a key risk factor in the development and maintenance of eating disorders (Stice, Gau, Rohde, & Shaw, 2017; Stice, Marti, & Durant, 2011) and depression (Farrer, Gulliver, Bennett, Fassnacht, & Griffiths, 2016). Devising treatment and prevention programs capable of addressing these negative outcomes requires a thorough understanding of the cognitive processes that underlie and maintain body dissatisfaction in women (Glashouwer, Jonker, Thomassen, & de Jong, 2016).

## Body dissatisfaction and attention

One approach to understanding body dissatisfaction focuses on cognitive processes, including how body-related information is perceived and attended. Cognitive theories of body dissatisfaction propose that schemas related to body shape and weight influence the processing of body-related stimuli, such that body-related stimuli attract attention and are preferentially processed relative to other stimuli (Vitousek & Hollon, 1990; Williamson, White, York-Crowe, & Stewart, 2004). Consistent with this view, Rodgers and DuBois (2016) reported an attentional maintenance bias in women with high body dissatisfaction, who tended to gaze at body-related stimuli longer and more frequently than stimuli that were not body-related. Women with attentional biases may be more vulnerable to continued body dissatisfaction due to the prevalence of body- and appearance-related stimuli in the media (Gao et al., 2014; Joseph et al., 2016; Rodgers & DuBois, 2016).

One study that examined the relationship between body satisfaction and attention used an eye-tracking paradigm to compare attention to images of “thin” and “fat” models in women who reported either high or low body dissatisfaction (Gao et al., 2014). Participants’ eye gaze was tracked during a free-viewing paradigm, in which participants viewed pairs of images over a 15-s presentation. The images included garden items, household items, women engaged in activities such as reading and talking, and either a thin or fat model image. Gao et al. found that body-dissatisfied women spent more time attending to both thin and fat models than body-satisfied women. They proposed that their results could be explained by body-dissatisfied women’s tendencies to make upward appearance comparisons. Fat model images, in contrast, may cue downward appearance comparisons, in which body-dissatisfied women associate their bodies with unattractive features in the fat models. Interestingly, and contrary to their hypotheses, participants’ state levels of body dissatisfaction did not increase after exposure to the thin and fat model images. This finding indicates that passively viewing such images during the eye-tracking task was not sufficient to activate negative body-related schemas.

Another recent study that also used a free-viewing eye-tracking paradigm to assess attention reported similar results (Tobin, Sears, Zumbusch, & von Ranson, 2018). Tobin et al. showed fat- and thin-related words to women with high or low body dissatisfaction. Displays of four words (one fat-related word, one thin-related word, and two neutral words) were presented for 8 s, and participants’ eye gaze was tracked while they viewed the words. Tobin et al. predicted that body-dissatisfied women would spend more time attending to fat-related words and less time attending to thin-related words compared to body-satisfied women. They instead found that body-dissatisfied women attended to both types of words more than body-satisfied women.

Tobin et al. (2018) also examined the effect of priming negative body-related schemas on attention to body-related words. Participants viewed a slideshow of 25 thin models, rating how closely each image matched their perception of the ideal female body. They found that the priming procedure increased state body dissatisfaction, but only for women in the high body dissatisfaction group. The priming did not affect attentional maintenance biases for either body-dissatisfied or body-satisfied women. This outcome is consistent with some studies that have used thin model exposure as a prime in body-dissatisfied women (Cassin, von Ranson, & Whiteford, 2008; Smith & Rieger, 2010), whereas other studies have reported an effect of thin model exposure on attentional maintenance (Johansson, Lundh, & Anderson, 2005; Labarge, Cash, & Brown, 1998; Lane, Mulgrew, Mahar, White, & Loughnan, 2017; Markis & McLennan, 2011). A possible reason for these mixed findings may be that the thin model exposure in some studies has not been potent enough to prime cognitive biases in body-dissatisfied women. One purpose of the present study was to determine whether a more elaborate priming procedure could affect body-dissatisfied women’s attentional biases.

Fewer studies have examined whether attentional biases related to body dissatisfaction can be reduced. Glashouwer, Jonker, Thomassen, and de Jong (2016) measured participants’ attentional biases to body parts perceived as “ugly” versus “beautiful” after 5 weeks of positive mirror exposure training. They found no changes in attention to “ugly” body parts among women with high body dissatisfaction, despite increases in their state body satisfaction. Loughnan, Mulgrew, and Lane (2015) found that an attention bias modification procedure designed to induce an attention bias to neutral words in a dot-probe task had no impact on participants’ state body satisfaction or attention to neutral versus appearance-related words. However, neither study examined whether laboratory-based priming procedures designed to increase state body satisfaction can reduce attention to body-related stimuli. Our study examined the malleability of attentional biases in body dissatisfied women by determining whether such biases can be modified by increasing state body satisfaction.

## The present study

The present study investigated whether attentional biases of women with body dissatisfaction could be influenced by body dissatisfaction priming and body satisfaction priming. An eye-tracking paradigm was used to measure attention to body images in women with high versus low body dissatisfaction. Like Gao et al. (2014), we used images of “thin” models, images of “fat” models, images of average-size women, and non-body images (household-related objects and garden-related objects). The priming procedures used in this study were designed to be more intensive than the procedures used in previous studies (e.g., Cassin et al., 2008; Smith & Rieger, 2010; Tobin et al., 2018). The priming tasks included a writing and reflection activity as well as viewing videos, all intended to increase or decrease body satisfaction. As described below, there were two main hypotheses examined: the first pertaining to the efficacy of the priming tasks and the second addressing the impact of priming on attention.

### Hypothesis 1: Priming effects on state body satisfaction and mood

We predicted that the priming tasks would be effective at modifying self-reported state body satisfaction, and specifically that (a) the body satisfaction priming procedure would increase participants’ state body satisfaction, regardless of their level of body dissatisfaction; and (b) the body dissatisfaction priming procedure would decrease participants’ state body satisfaction, but only for women with high body dissatisfaction (as found by Tobin et al., 2018). We also predicted that the priming tasks would affect participants’ mood, and specifically, that (c) the body satisfaction priming task would increase participants’ self-rated mood and (d) the body dissatisfaction priming task would decrease participants’ self-rated mood. Measuring changes in mood due to priming provided additional evidence on the effectiveness of the priming tasks (e.g., Bessenoff, 2006; Brown & Tiggemann, 2016; Tiggemann, Polivy, & Hargreaves, 2009).

### Hypothesis 2: Priming effects on attention

The major purpose of our study was to determine how changes in body satisfaction produced by priming body satisfaction and body dissatisfaction are reflected in attention. Our predictions were (a) prior to the priming procedures, women with high body dissatisfaction would attend to thin and fat model images more than women with low body dissatisfaction; (b) the body satisfaction priming procedure would decrease attention to body images among women with high body dissatisfaction relative to their pre-priming level of attention, whereas for women with low body dissatisfaction, the priming would have no effect on their attention to body images; and (c) the body dissatisfaction priming procedure would increase attention to body images among women with high body dissatisfaction relative to their pre-priming level of attention, whereas for women with low body dissatisfaction, the priming would have no effect.

## Method

The study involved two phases. First, eligible participants for the eye-tracking procedure were identified via an online survey. Second, eligible participants visited the laboratory to provide eye-tracking data.

### Measures

#### Body Shape Questionnaire

The Body Shape Questionnaire (BSQ; Cooper, Taylor, & Fairburn, 1987) consists of 34 questions that assess body shape concerns over the past 4 weeks (e.g., “Has worry about your shape made you diet?”). Each item is rated on a 6-point Likert-type scale, with 1 representing “never” and 6 representing “always.” Higher total scores indicate greater trait body dissatisfaction, with scores less than 80 indicating no body concerns (Taylor, 1987). The BSQ has high internal consistency (Rosen, Jones, Ramirez, & Waxman, 1996). Alpha for the current sample was .97.

#### Body Appreciation Scale

The Body Appreciation Scale (BAS-2; Tylka & Wood-Barcalow, 2015) assesses attitudes of acceptance and respect for the body (e.g., “I feel that my body has at least some good qualities”). The measure consists of 10 questions, each rated on a 5-point Likert-type scale, with 1 representing “never” and 5 representing “always.” Participants’ scores on the 10 items are averaged to create an overall score, with higher scores indicating higher body appreciation. The BAS-2 has been found to have high internal consistency for women and is strongly negatively correlated with body dissatisfaction (Tylka & Wood-Barcalow, 2015). BAS-2 scores predict unique variance in intuitive eating, self-esteem, and proactive coping (for men and women), and for women, BAS-2 scores also predict unique variance in eating disorders symptomatology (Tylka & Wood-Barcalow, 2015). Consistent with Tylka and Wood-Barcalow observations, in the current sample, the correlation between the BAS-2 and BSQ scores was −.73 (*N* = 108, *p* < .001), indicating that these constructs are strongly related (with 57% shared variance), yet distinct. Alpha for the current sample was .92.

#### Body satisfaction visual analogue scale

Participants’ state level of body satisfaction was measured using a visual analog scale (VAS). The body satisfaction VAS consisted of a 100-mm horizontal line, with the left side labeled “very dissatisfied” and the right side labeled “very satisfied.” Participants were asked to rate their current body satisfaction by placing a mark on the line. This mark was assigned a score from −50 to +50 by measuring its distance in millimeters from the midpoint of the line. This measure was used successfully by Tobin et al. (2018) to measure changes in state body satisfaction after a thin model priming task, and similar scales have been used in previous studies of priming effects on state body satisfaction (e.g., Gao et al., 2014; Glashouwer et al., 2016). Participants completed a body satisfaction VAS before and after each new task and placed the scale in an envelope to keep their responses private. This procedure was designed to minimize demand characteristics associated with the priming manipulation.

#### Mood visual analogue scale

Participants’ mood state was assessed using a VAS. The mood VAS consisted of a 100-mm horizontal line, with the left side labeled “very negative” and the right side labeled “very positive.” Participants rated their current mood by placing a mark on the line. This mark was assigned a score from −50 to +50 by measuring its distance in millimeters from the midpoint of the line, such that negative scores reflect negative mood and positive scores reflect positive mood. Similar VAS scales have been used to measure mood in a wide variety of studies (e.g., Frayn, Sears, & von Ranson, 2016; Scherrer & Dobson, 2015). Participants completed the mood VAS before and after each new task.

#### Body mass index

Body mass index (BMI, kg/m^2^) is an estimate of body adiposity (Romero-Corral et al., 2008). Participants self-reported their heights and weights and their BMIs were calculated from these data. Self-reported height and weight tend to correlate highly with measured height and weight (*r* = .98 and *r* = .99, respectively; Pursey, Burrows, Stanwell, & Collins, 2014).

### Priming tasks

Assignment to the body dissatisfaction and body satisfaction priming tasks was counterbalanced across participants. The priming tasks were intended to be similar in length and content, such that both priming tasks involved watching videos and a writing activity and each required approximately 10 min to complete. For the body dissatisfaction prime, participants first watched a 5-min video of a Sports Illustrated photo shoot of swimsuit models (Sports Illustrated Swimsuit, 2016, 2017). This video was chosen for its similarity to typical body dissatisfaction priming tasks involving slideshows of thin model images. After watching the video, participants were asked to complete a writing activity in a private room. Participants wrote on a sheet of paper that included the instructions:

For the next five minutes, imagine that you are standing in a room surrounded by mirrors, viewing your own nude body. With this image in mind, write about the parts of your body that you dislike or wish you could change, and why. Next, imagine that another person is standing in the room, evaluating your body, and describe the specific aspects of your body you think this person would evaluate negatively, and why. Be as specific as you can. You can choose to keep your writing or have it confidentially shredded. The researcher will not read anything you have written.

For the body satisfaction prime, participants watched three short videos encouraging body acceptance, totaling approximately 5 min (BuzzFeedYellow, 2015; I Heart My Body, 2016; Upworthy, 2016). The videos presented a variety of positive body image attitudes, including celebration of body diversity, appreciation for body functionality, and love for the body. Participants were then given 5 min to complete a writing activity in a private room. Participants wrote on a sheet of paper that included the instructions:

For the next five minutes, think about the aspects of your body that you are most proud of. For example, you might take pride in certain features of your body or physical appearance, or you might be proud of things your body has allowed you to do. Write down as many specific aspects as you can think of that make you feel pride or respect for your body, and why. Be as specific as you can. You can choose to keep your writing or have it confidentially shredded. The researcher will not read anything you have written.

For ethical reasons, participants who were assigned to the body dissatisfaction priming task completed the body satisfaction priming task before leaving the laboratory.

### Stimuli for eye-tracking

Five categories of images were used to create the eye-tracking displays: images of thin models, images of fat models, images of average women, images of household-related objects, and images of gardening-related objects. All the images were collected from the Internet. These categories were similar to those used by Gao et al. (2014), although a different set of images was used.

To identify an optimal set of images, an online survey using Qualtrics (www.qualtrics.com) was used to collect ratings for a large number of images. Female undergraduate students (*N* = 100) completed the ratings in exchange for bonus credit in a psychology course. None of these students participated in the eye-tracking phase of the study. The survey included 45 fat model images that showed women with larger bodies wearing either underwear or bathing suits, 45 thin model images that showed women with smaller bodies wearing either underwear or bathing suits, and 90 images of women with average-sized bodies engaged in various activities (e.g., reading, talking, walking, working on a computer; in these images, body shape and weight were much less salient). The images of women with average bodies served as control stimuli given that these images included women but did not focus on women’s weight or body shape. The survey also included 70 gardening-related images (e.g., flowers, gardening tools) and 70 household-related images (e.g., furniture, kitchen tools). There were a total of 320 images to be rated, and each participant was assigned a randomly-selected one third of these images to rate (106 to 107 images). All the images were rated for valence, using a scale from −3 (“extremely negative”) to +3 (“extremely positive”). The three categories of images that included pictures of women (fat model images, thin model images, images of average women) were rated for body size, using a scale from −3 (“extremely thin”) to +3 (“extremely fat”), with a midpoint of 0 (“neutral”). These images were also rated for attractiveness, using a scale from −3 (“extremely unattractive”) to +3 (“extremely attractive”), with a midpoint of 0 (“neutral”).

Using the body size ratings, the 32 thin model images with the lowest average rating of body size (i.e., those closest to a rating of “extremely thin”) were selected for the study. Similarly, the 32 fat model images with the highest average rating of body size (i.e., those closest to a rating of “extremely fat”) were selected. For the images of average women, the 64 images with ratings closest to 0 (“neutral”) were selected. Sixty-four gardening-related images and 64 household-related images were randomly selected.

### Eye-tracking apparatus

Eye movements were recorded using an EyeLink 1000 eye-tracking system (SR Research Ltd, Ottawa, Ontario, Canada), which uses infrared video-based tracking technology. The system has a 1,000-Hz sampling rate, a temporal resolution of 2 ms, and an average gaze error of less than .5° of visual angle. Stimuli were shown on a 24-in. LCD monitor positioned approximately 60 cm away from the participant. Participants used a chin rest to minimize head movements while they viewed the images to maximize tracking accuracy.

### Participants

Prospective participants were identified using an online survey completed in a computer lab and administered via Qualtrics. Female undergraduate students (*N* = 525) completed the survey in small groups and received bonus credit in a psychology course in exchange for their participation. The survey included the BSQ (Cooper et al., 1987), the BAS-2 (Tylka & Wood-Barcalow, 2015), and demographic questions including age, ethnicity, and education. Participants also provided their height and weight so their BMI could be calculated. After excluding participants with numerous missing responses and those who were not interested in a laboratory visit, 457 eligible participants remained. Participants were grouped into top, middle, and bottom tertiles based on their total BSQ score, using cutoffs developed by Tobin et al. (2018). There were 192 participants in the top tertile (total BSQ score > 104), 156 in the middle tertile (total BSQ score ranging from 73 to 103), and 109 in the bottom tertile (total BSQ score < 72). Only women in the top tertile (those with high trait body dissatisfaction) and the bottom tertile (those with low trait body dissatisfaction) were invited to participate in the laboratory visit.

Eligible participants were invited via e-mail for a laboratory visit. Participants received additional bonus credit in a psychology course for participating. Participation rates of those contacted were 47.7% for women in the top tertile and 50.5% for women in the bottom tertile. Women who declined to participate in the study were similar to those who participated; for example, they were similar in age (*M* = 20.40 years vs. *M* = 20.98), *t*(240) = 0.92, *p* = .35, and BMI (*M* = 23.29 vs. *M* = 22.68 kg/m^2^), *t*(242) = 0.26, *p* = .79. χ^2^ analyses indicated there were no differences in ethnicity or education level (*p*s > .10), and *t*-tests indicated there were no differences in scores on the BSQ or BAS (*p*s > .10). The absence of such differences suggests that there was no obvious selection bias that could have influenced the results.

Eye-tracking data were collected from 108 women, 65 with BSQ scores in the top tertile (women with high trait body dissatisfaction) and 43 with BSQ scores in the bottom tertile (women with low trait body dissatisfaction). Descriptive statistics for the groups are listed in Table 1. The groups did not differ significantly in age or ethnicity (*p*s > .10). As expected, women with high body dissatisfaction had significantly higher BSQ scores (*M* = 129.00) than women with low body dissatisfaction (*M* = 60.01). Consistent with this difference, women with high body dissatisfaction had significantly lower pre-prime body satisfaction VAS ratings (*M* = 1.15) than women with low body dissatisfaction (*M* = 27.33). Women with high body dissatisfaction also had lower BAS scores (*M* = 3.00) than women with low body dissatisfaction (*M* = 4.11). The groups did not differ in their pre-prime mood VAS ratings.

**Table 1.** Descriptive statistics for the low and high body dissatisfaction groups.

	Low body dissatisfaction			High body dissatisfaction			*t*/χ^2^
	*M*	*SD*	Range	*M*	*SD*	Range	
Age	21.07	3.86	18–37	20.92	4.16	17–39	0.18
Body mass index (kg/m^2^)	20.93	2.83	13.08–29.99	23.85	4.43	17.99–44.99	3.82*
Caucasian (%)	48.83	—	—	47.69	—	—	0.01
Body Shape Questionnaire	60.01	10.20	36–72	129.00	18.78	104–182	22.01*
Pre-prime body satisfaction VAS	27.33	12.48	1–49	1.15	17.06	−42–30	8.63*
Pre-prime mood VAS	22.47	18.23	−13–50	18.02	17.58	−25–50	1.26
Body Appreciation Scale	4.11	0.53	3.0–5.0	3.00	0.80	1.0–4.2	7.93*

*Note.* VAS = visual analogue scale. Significant between-group differences at *p* < .05 in a *t*-test or χ^2^ comparison, as appropriate, are indicated (*).

### Procedure

Participants were provided with verbal and written instructions outlining the study procedure at the beginning of their laboratory visit. After providing informed consent, participants completed baseline mood VAS and state body satisfaction VAS measures. The eye-tracking data were collected in a small room dedicated to this purpose. The eye-tracking system was first calibrated for the participant, a procedure that required approximately 5 min. Data collection began once the calibration was successful.

**Figure 1.**
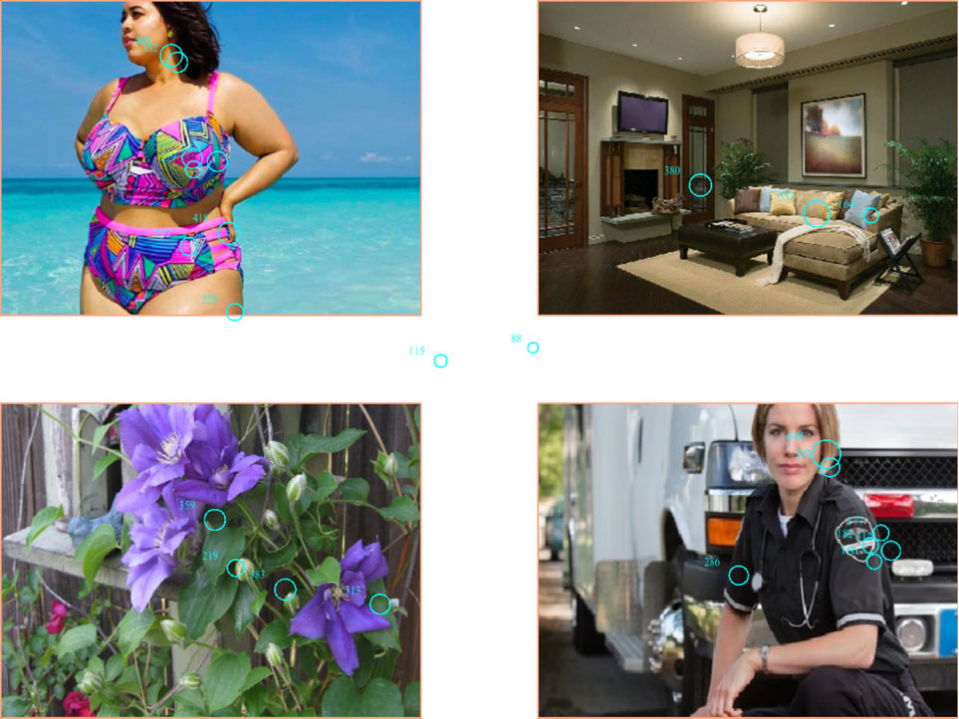
An example display with eye-tracking data. The small circles denote individual fixations; numbers adjacent to fixations indicate the duration of the fixation (in milliseconds). The fixations are superimposed on the display for illustration purposes and were not visible to participants.

At the start of each trial, the participant fixated on a black dot in the center of the display to ensure proper gaze measurement. Each trial consisted of the presentation of four images simultaneously, with one image presented in each of the four quadrants of the display. An example display is shown in Figure 1. Each display consisted of one household-related image, one gardening-related image, one image of an average woman, and either a thin or a fat model image. The placement of images in the display was counterbalanced to ensure that each image type was equally likely to appear in each quadrant. The images were the same size and care was taken to match them on color, brightness, and complexity. Each display was presented for 8 s, and the participant’s eye gaze was tracked and recorded throughout this interval. Participants were instructed to look at the images freely as if they were watching a slide show. There were two practice trials followed by 32 experimental trials. Viewing all the images required approximately 5 min.

After viewing the images, participants completed the body satisfaction and mood VAS measures, followed by either the body satisfaction or body dissatisfaction priming task. State body satisfaction and mood VAS measures were completed again immediately after the priming task. Participants then viewed a new set of images so that post-priming eye-tracking data could be collected. Like the first displays, these displays consisted of one household-related image, one gardening-related image, one image of an average woman, and either a thin or a fat model image. The order of presentation of the two sets of images was counterbalanced across participants. There were two practice trials followed by 32 experimental trials. Following the second eye-tracking task, participants completed state body satisfaction and mood scale VAS measures. Participants then completed the BSQ and a final set of state body satisfaction and mood scale VAS measures. All participants were debriefed at the end of the session.

## Results

To assess the efficacy of the two priming tasks, the analyses of the body satisfaction VAS ratings are described first, followed by the analyses of the participants’ mood ratings. The analyses of the eye-tracking data are then described; these were designed to identify group differences in attention to the thin and fat model images before and after the priming procedures. All statistical analyses were carried out using the Statistical Package for the Social Sciences (SPSS, version 25).

### Hypothesis 1: Priming effects on state body satisfaction and mood

Participants’ state body satisfaction VAS ratings measured before and after priming were analyzed to determine whether there was an effect of priming task on state body dissatisfaction and whether this effect differed depending on trait body dissatisfaction. The ratings were analyzed using a mixed-model analysis of variance (ANOVA), with the factors Body Dissatisfaction Group (women with low body dissatisfaction, women with high body dissatisfaction) and Time of Measurement (baseline, pre-prime, post-prime 1, post-prime 2, post-prime 3). The post-prime measurement 1 was collected after the body satisfaction/dissatisfaction prime, the post-prime measurement 2 was collected after the second eye-tracking procedure, and the post-prime measurement 3 was collected at the end of the testing session. Data from the body satisfaction priming task and the body dissatisfaction priming task were analyzed separately given that different predictions were made for the two priming tasks.

**Figure 2.**
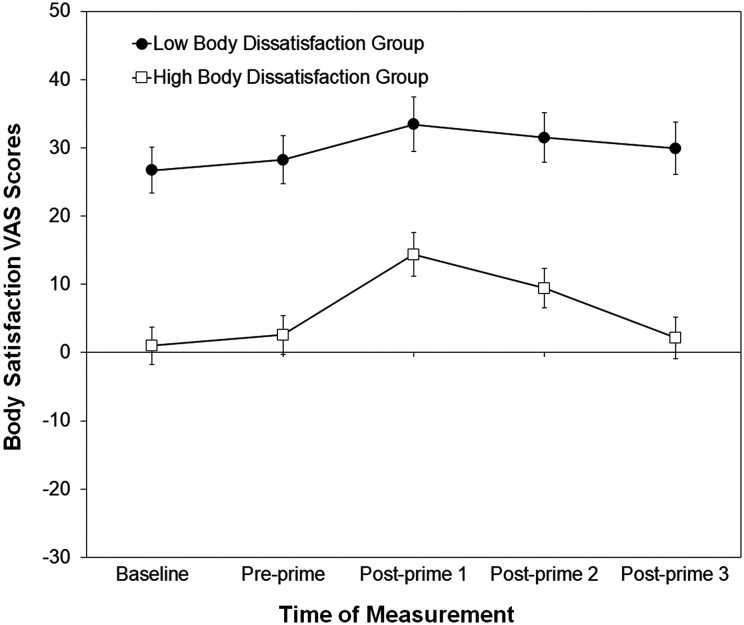
Mean body satisfaction VAS scores for women assigned to the body satisfaction priming task, measured at baseline, pre-prime, and post-prime (post-prime 1 = after the body satisfaction prime, post-prime 2 = after second eye-tracking data collection, and post-prime 3 = end of testing session). Error bars represent one standard error of the mean. VAS = visual analog scale.

Figure 2 shows the mean ratings collected in the body satisfaction priming task from women in the high and low body dissatisfaction groups. As expected, there was a main effect of Body Dissatisfaction Group, *F*(1, 52) = 39.29, *p* < .001, ${\text{η}}_{\text{p}}^{2}$ = .430. That is, overall, women with high body dissatisfaction had lower state body satisfaction VAS ratings (*M* = 5.89) than women with low body dissatisfaction (*M* = 29.95). There was also a main effect of Time of Measurement, *F*(4, 208) = 7.64, *p* < .001, ${\text{η}}_{\text{p}}^{2}$ = .128, reflecting variation in the ratings across the different measurement times. The interaction between Body Dissatisfaction Group and Time of Measurement was not significant, *F*(4, 208) = 1.33, *p* = .258. The absence of an interaction indicated that the pattern of changes in state body satisfaction ratings was similar for women in the high and low body dissatisfaction groups. As can be seen in Figure 2, state body satisfaction ratings for both groups increased following the body satisfaction priming (pre-prime vs. post-prime 1), which indicates that the priming was effective for both groups of women (confirming hypothesis 1a).

**Figure 3.**
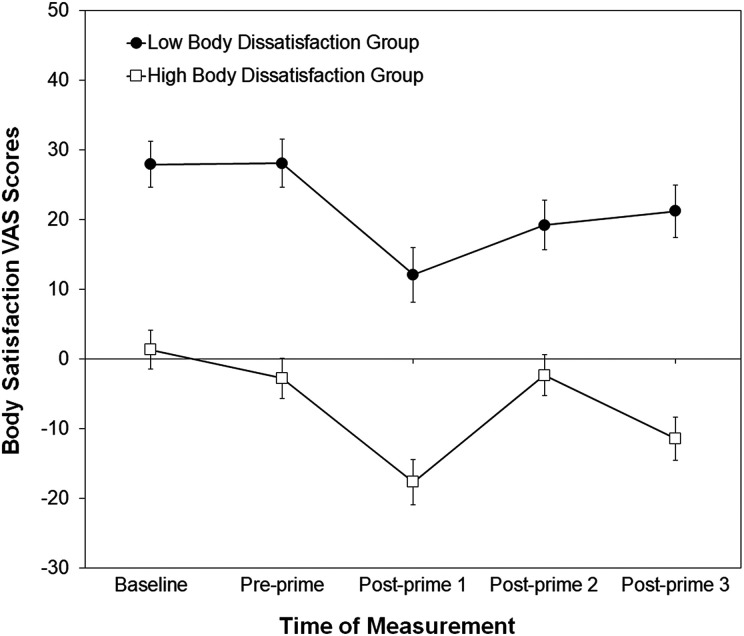
Mean body satisfaction VAS scores for women assigned to the body dissatisfaction priming task, measured at baseline, pre-prime, and post-prime (post-prime 1 = after the body dissatisfaction prime, post-prime 2 = after second eye-tracking data collection, and post-prime 3 = end of testing session). Error bars represent one standard error of the mean. VAS = visual analog scale.

Figure 3 shows the mean ratings collected in the body dissatisfaction priming task. The ANOVA revealed the expected main effect of Body Dissatisfaction Group, *F*(1, 52) = 47.56, *p* < .001, ${\text{η}}_{\text{p}}^{2}$ = .478. Overall, women with high body dissatisfaction had lower state body satisfaction VAS ratings (*M* = −6.59) than women with low body dissatisfaction (*M* = 21.68). There was also a main effect of Time of Measurement, *F*(4, 208) = 27.89, *p* < .001, ${\text{η}}_{\text{p}}^{2}$ = .349, and an interaction, *F*(4, 208) = 2.76, *p* = .029, ${\text{η}}_{\text{p}}^{2}$ = .050. As can be seen in Figure 3, mean state body satisfaction ratings for both groups decreased following the body dissatisfaction priming (pre-prime vs. post-prime 1). For women with high body dissatisfaction, the difference in state body satisfaction ratings pre-prime versus post-prime (2.80 vs. −17.66) was significant, *t*(31) = 5.99, *p* < .001, which was also true for women with low body dissatisfaction (28.05 vs. 12.09), *t*(21) = 5.34, *p* < .001 (disconfirming hypothesis 1b). The interaction reflected group differences in the pattern of ratings post-prime, which were similar but not identical, although for both groups of women state body satisfaction ratings collected at the end of the study (post-prime 3) were significantly higher (*p*s < .05) than those collected immediately after the prime (post-prime 1). Taken together, these analyses show that both the body satisfaction and dissatisfaction priming tasks changed participants’ ratings of state body satisfaction, for women in both the high and low body dissatisfaction groups: the body satisfaction priming task increased participants’ state body satisfaction, whereas the body dissatisfaction priming task decreased participants’ state body satisfaction.^1^

To determine if the body satisfaction and body dissatisfaction priming tasks influenced participants’ mood in the predicted directions (increases in positive mood following the body satisfaction priming and decreases in positive mood following the body dissatisfaction priming), the mood ratings (measured on a scale from −50 to +50) were analyzed using a mixed-model ANOVA, with the factors Body Dissatisfaction Group (women with low body dissatisfaction, women with high body dissatisfaction) and Pre- versus Post-prime (pre-prime, post-prime). Data from the body satisfaction and body dissatisfaction priming tasks were analyzed separately given the different predictions made for the two priming tasks (hypothesis 1c and 1d).

For the body satisfaction priming task, there was a main effect of Body Dissatisfaction Group, *F*(1, 52) = 10.85, *p* < .001, ${\text{η}}_{\text{p}}^{2}$ = .173, with women with high body dissatisfaction having lower mood ratings overall (*M* = 15.62) than women with low body dissatisfaction (*M* = 27.28). As predicted (hypothesis 1c), there was a main effect of Pre- versus Post-prime, *F*(1, 52) = 9.92, *p* = .003, ${\text{η}}_{\text{p}}^{2}$ = .160, as mood ratings increased pre-prime versus post-prime (*M* = 17.74 vs. 25.11). There was no interaction, *F*(1, 52) = 0.92, *p* = .341, ${\text{η}}_{\text{p}}^{2}$ = .017, which indicated that the body satisfaction priming had similar effects on the mood of women with high body dissatisfaction (*M* = 13.06 vs. 18.18, pre- vs. post-prime) and the mood of women with low body dissatisfaction (*M* = 22.42 vs. 32.04, pre- vs. post-prime).

For the body dissatisfaction priming task, the main effect of Body Dissatisfaction Group was significant, *F*(1, 52) = 4.20, *p* = .045, ${\text{η}}_{\text{p}}^{2}$ = .075, with women with high body dissatisfaction having lower mood ratings overall (*M* = 6.64) than women with low body dissatisfaction (*M* = 15.90). There was also a main effect of Pre- versus Post-prime, *F*(1, 52) = 28.80, *p* < .001, ${\text{η}}_{\text{p}}^{2}$ = .356. Mood ratings decreased following the body dissatisfaction priming (*M* = 16.29 vs. 6.25, pre-prime vs. post-prime), confirming hypothesis 1d. There was no interaction, *F*(1, 52) = 1.65, *p* = .204, ${\text{η}}_{\text{p}}^{2}$ = .031. The decrease in mood ratings due to the body dissatisfaction priming was similar for women with high body dissatisfaction (*M* = 12.87 vs. 0.42, pre- vs. post-prime) and women with low body dissatisfaction (*M* = 19.72 vs. 12.09, pre- vs. post-prime), hence the absence of an interaction. These analyses indicate that the body satisfaction and body dissatisfaction priming tasks influenced participants’ mood in the expected directions and are additional evidence of the efficacy of the priming procedures.

### Hypothesis 2: Priming effects on attention

The fixation data were processed using the EyeLink Data Viewer analysis software (SR Research) to filter for blinks, missing data, and other recording artifacts (using the default settings). To be included in the analyses, a fixation had to be at least 100 ms in duration; adjacent, sequential fixations less than 100 ms were merged into a single fixation. For the analyses, the dependent variable was the total fixation time to an image during the 8-s presentation (i.e., the sum of all fixation durations). Longer total fixation times reflect greater attention to an image. This value was calculated for each image type (thin model images, fat model images, images of average women) within a trial and then averaged over the 32 trials. To simplify the analyses, total fixation times to gardening-related images and household-related images were averaged together and are hereafter referred to as neutral images.

#### Hypothesis 2a: Group differences in attention prior to priming

**Figure 4.**
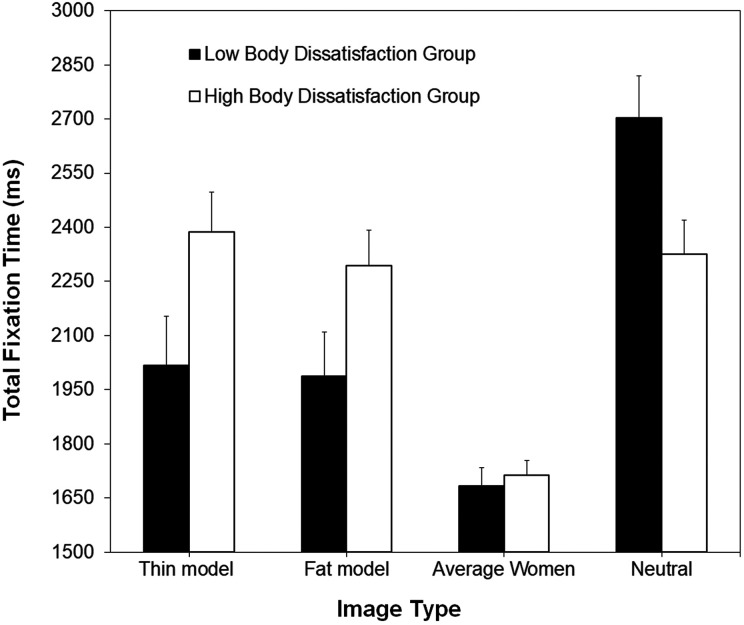
Mean total fixation times (in milliseconds) for thin model images, fat model images, images of average women, and neutral images, for women in the high and low body dissatisfaction groups. These data were collected before the priming task. Error bars represent one standard error of the mean.

We first analyzed the pre-prime fixation data to determine whether women in the high and low body dissatisfaction groups differed in their attention to the images before they experienced the body satisfaction or body dissatisfaction priming. These data were analyzed using a mixed-model ANOVA, with the factors Body Dissatisfaction Group (women with low body dissatisfaction, women with high body dissatisfaction) and Image Type (thin model images, fat model images, images of average women, neutral images). Figure 4 shows the results for both groups of women. The main effect of Body Dissatisfaction Group was significant, *F*(1, 106) = 4.09, *p* = .046, ${\text{η}}_{\text{p}}^{2}$ = .037, as was the main effect of Image Type, *F*(3, 318) = 17.81, *p* < .001, ${\text{η}}_{\text{p}}^{2}$ = .14. More important, there was a significant interaction between Body Dissatisfaction Group and Image Type, *F*(3, 318) = 4.55, *p* = .004, ${\text{η}}_{\text{p}}^{2}$ = .041. As can be seen in Figure 4, the interaction indicated that women with high body dissatisfaction differed from women with low body dissatisfaction in their attention to thin model and fat model images, but not to images of average women. More specifically, women with high body dissatisfaction had longer total fixation times to thin model images (*M* = 2,388 ms vs. *M* = 2,018 ms) and fat model images (*M* = 2,293 ms vs. 1,988 ms) than women with low body dissatisfaction, *t*(106) = 2.12, *p* = .036; *t*(106) = 1.93, *p* = .055, respectively, whereas for images of average women, there was no difference (*M* = 1,713 ms vs. 1,683 ms), *t*(106) = 0.45, *p* = .647. These results indicate that the women with high body dissatisfaction exhibited an attentional bias to thin and fat model images prior to the priming procedure, replicating the findings of Gao et al. (2014) and confirming hypothesis 2a.

#### Hypotheses 2b and 2c: Effect of priming procedures on attention

To assess the effect of the priming procedures, all of the fixation data were first analyzed using a four-factor mixed-model ANOVA, with the factors Body Dissatisfaction Group (women with low body dissatisfaction, women with high body dissatisfaction), Priming Task (body satisfaction priming, body dissatisfaction priming), Image Type (thin model images, fat model images, images of average women, neutral images), and Pre- versus Post-prime (pre-prime, post-prime). There were main effects of Body Dissatisfaction Group, *F*(1, 104) = 5.92, *p* = .017, ${\text{η}}_{\text{p}}^{2}$ = .054; Priming Task, *F*(1, 104) = 4.95, *p* = .028, ${\text{η}}_{\text{p}}^{2}$ = .045; Image Type, *F*(3, 312) = 18.40, *p* < .001, ${\text{η}}_{\text{p}}^{2}$ = .150; and Pre- versus Post-prime, *F*(1, 104) = 19.84, *p* < .001, ${\text{η}}_{\text{p}}^{2}$ = .16. There were also two-way interactions between Body Dissatisfaction Group and Image Type, *F*(3, 312) = 6.15, *p* < .001, ${\text{η}}_{\text{p}}^{2}$ = .056; Priming Task and Image Type, *F*(3, 312) = 4.41, *p* = .005, ${\text{η}}_{\text{p}}^{2}$ = .041; and Pre- versus Post-prime and Image Type, *F*(3, 312) = 9.22, *p* < .001, ${\text{η}}_{\text{p}}^{2}$ = .082. The highest order interaction was the three-way interaction among Priming Task, Image Type, and Pre- versus Post-prime, *F*(3, 312) = 3.49, *p* = .016, ${\text{η}}_{\text{p}}^{2}$ = .033. Importantly, the four-way interaction among Body Dissatisfaction Group, Priming Task, Image Type, and Pre- versus Post-prime was not significant, *F*(3, 312) = 0.59, *p* = .620. The absence of a four-way interaction indicates that the body satisfaction and body dissatisfaction priming tasks had similar effects for women in the high and low body dissatisfaction groups. This was confirmed in separate analyses of the priming tasks: for both the body satisfaction and body dissatisfaction priming tasks, there was no three-way interaction among Body Dissatisfaction Group, Image Type, and Pre- versus Post-prime (*p* = .47 and *p* = .94, respectively). This three-way interaction would have been present had the priming effects differed for the two groups. The follow-up analyses described below therefore collapsed across body dissatisfaction group.

To follow up the highest order interaction among Priming Task, Image Type, and Pre- versus Post-prime, the data from the body satisfaction and body dissatisfaction priming tasks (collapsed across body dissatisfaction group) were analyzed separately (Image Type × Pre- vs. Post-prime ANOVAs). For the body satisfaction priming task, there was a main effect of Image Type, *F*(3, 159) = 9.24, *p* < .001, ${\text{η}}_{\text{p}}^{2}$ = .149; a main effect of Pre- versus Post-prime, *F*(1, 53) = 4.49, *p* = .039, ${\text{η}}_{\text{p}}^{2}$ = .078; and no interaction, *F*(3, 159) = 1.08, *p* = .356. Follow-up comparisons using *t*-tests indicated that total fixation times to thin model images were not significantly different pre- versus post-prime (*M* = 2,080 ms vs. 2,167 ms), *t*(53) = 0.92, *p* = .359. This was also the case for fat model images (*M* = 2,108 ms vs. 2,240 ms), *t*(53) = 1.54, *p* = .128, and images of average women (*M* = 1,715 ms vs. 1,742 ms), *t*(53) = 0.62, *p* = .532. These results indicate that the body satisfaction priming did not affect attention to body images, contrary to the prediction that the priming would decrease attention to body images for women with high body dissatisfaction (hypothesis 2b). Note that the absence of statistically significant priming effects cannot be attributed to low statistical power; power was more than adequate to detect a “medium” effect size (Cohen’s *d* = .50) given that the priming effect was measured within-subjects (power was calculated to be 97% for a medium effect size, using G*Power 3.1; Faul, Erdfelder, Lang, & Buchner, 2007).

**Figure 5.**
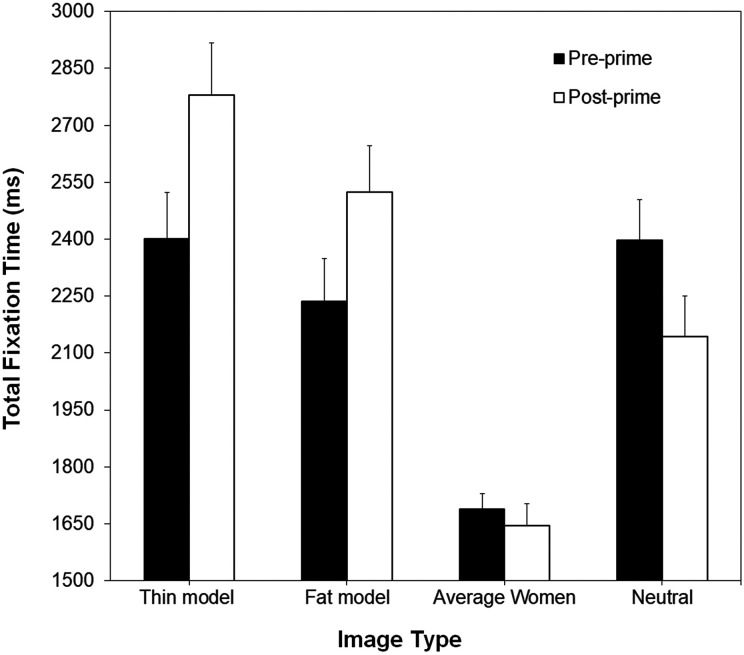
Mean total fixation times (in milliseconds) for thin model images, fat model images, images of average women, and neutral images, for the body dissatisfaction priming task. These data are collapsed across the high and low body dissatisfaction groups. Error bars represent one standard error of the mean.

For the body dissatisfaction priming task, there was a different pattern of results. There was a main effect of Image Type, *F*(3, 159) = 12.77, *p* < .001, ${\text{η}}_{\text{p}}^{2}$ = .194; a main effect of Pre- versus Post-prime, *F*(1, 53) = 21.40, *p* < .001, ${\text{η}}_{\text{p}}^{2}$ = .288; and a significant interaction, *F*(3, 159) = 13.89, *p* < .001, ${\text{η}}_{\text{p}}^{2}$ = .207. These results are shown in Figure 5. *t*-tests used to follow up the interaction revealed that total fixation times to thin model images increased after the body dissatisfaction priming (*M* = 2,401 ms vs. 2,779 ms), *t*(53) = 4.75, *p* < .001, as did total fixation times to fat model images (*M* = 2,236 ms vs. 2,524 ms), *t*(53) = 3.21, *p* = .002 (the effect sizes of these differences were *d* = .61 and *d* = .52, respectively). For images of average women, on the other hand, there was no difference in total fixation times pre- versus post-priming (*M* = 1,688 ms vs. 1,646 ms), *t*(53) = 0.95, *p* = .344. Thus, the body dissatisfaction priming, unlike the body satisfaction priming, altered women’s attention to fat and thin model images. However, contrary to our prediction (hypothesis 2c), this effect was the same for women with high and low body dissatisfaction (as evidenced by the lack of interactions with Body Dissatisfaction Group).^2^

## Discussion

The purpose of this study was to examine whether priming tasks designed to increase state body satisfaction and state body dissatisfaction would influence attentional biases to body images among women with high and low trait body dissatisfaction. Women with high and low levels of body dissatisfaction, as measured by the BSQ (Cooper et al., 1987), were randomly assigned to a body satisfaction or body dissatisfaction priming task, and eye-tracking data were collected before and after the priming. Our study contributes to researchers’ understanding of attentional biases in women with body dissatisfaction by determining whether the biases can be affected by priming manipulations.

### Priming effects on state body satisfaction and mood

As predicted, women with high trait body dissatisfaction were affected by both the body satisfaction and body dissatisfaction priming tasks, as their self-reported state body satisfaction and mood increased or decreased post-priming, respectively. Contrary to our prediction, the state body satisfaction of women with low trait body dissatisfaction (i.e., those who were relatively satisfied with their bodies) was also affected by the priming tasks, and in a manner very similar to the women with high body dissatisfaction. This outcome diverges from the findings of Tobin et al. (2018), who reported that their body dissatisfaction priming task did not affect the body satisfaction ratings of women with low body dissatisfaction. However, the priming task used by Tobin et al. involved a brief slideshow of thin model images, and they speculated that it might not have been potent enough to affect state body satisfaction in women with low trait body dissatisfaction. An advantage of our study was the use of a lengthier and more engaging priming procedure, in which participants were asked to write about aspects of their body they disliked after viewing a video of a swimsuit model photo shoot. As women with low trait body dissatisfaction were susceptible to this priming, we conclude that women with low levels of body dissatisfaction are vulnerable to body dissatisfaction cues in their environment. Importantly, we found that women with low body dissatisfaction were also responsive to the positive body image messaging incorporated into the body satisfaction priming task.

### Priming effects on attention

Consistent with Gao et al.’s (2014) findings and our prediction, prior to the priming tasks, women with high body dissatisfaction attended to thin and fat model images more than women with low body dissatisfaction. This result replicates the findings of other investigators who have found that women with negative self-schemas related to weight exhibit attentional biases for both fat- and thin-related information (Rodgers & DuBois, 2016; Tobin et al., 2018; Vitousek & Hollon, 1990; Williamson et al., 2004). Also consistent with predictions was our finding that women with high body dissatisfaction increased their attention to thin and fat model images following the body dissatisfaction priming. In this respect, our results differ from those of Gao et al. (2014) and Tobin et al. (2018), who also used body dissatisfaction priming but did not find that it changed the attentional biases of women with high (or low) body dissatisfaction. Again, this difference is likely due to the greater intensity and efficacy of the body dissatisfaction priming task we used.

Another unexpected finding was that women with low body dissatisfaction increased their attention to thin and fat model images after the body dissatisfaction priming. This outcome indicates that women with low body dissatisfaction are not immune to the negative effects of body dissatisfaction priming. Indeed, our results showed that the priming had a very similar effect on attention for women with low body dissatisfaction and women with high body dissatisfaction (i.e., increased attention to thin and fat model images). Once again, the more elaborate body dissatisfaction priming procedure used in the present study likely accounts for this discrepancy.

In contrast to body dissatisfaction priming, we found that body satisfaction priming did not affect attention to body-related images for women with either high or low body dissatisfaction, even though the task was effective at increasing state body satisfaction (as measured by the body satisfaction VAS). This finding suggests that body satisfaction priming has a negligible ability to affect women’s attentional biases, or, alternatively, that the body satisfaction priming task was not potent enough to affect attentional biases. One should keep in mind that the body satisfaction priming task necessarily brought participants’ own bodies to the forefront of their attention, given that they were asked to write about aspects of their body they liked or were proud of. This focus on the body may have paradoxically resulted in heightened attention to perceived imperfections and may have reduced positive effects of the priming procedure. We do know that the body satisfaction priming task was not as effective as the body dissatisfaction priming task as measured by the body satisfaction VAS ratings. That is, comparing the percentage change in state body satisfaction pre- versus post-priming, the change was smaller for the body satisfaction priming task. Specifically, for the body satisfaction priming task, the increase in state body satisfaction VAS ratings (averaged over the two groups) was 55% (15.41 vs. 23.90), whereas for the body dissatisfaction priming task, the decrease in state body satisfaction VAS ratings was 122% (12.62 vs. −2.78). Thus, it is possible that the difference between the two priming tasks in their ability to affect attention was partially or entirely due to this difference in their efficacy, and that a different type of body satisfaction priming could produce changes in women’s attentional biases (e.g., a procedure that does not focus attention on the body). This possibility will be an important consideration for future research.

### Limitations, directions for future research, and clinical implications

This study had several limitations that should be considered when interpreting its findings. First, nonclinical samples of undergraduate women were recruited for the study, and therefore our findings cannot be generalized to clinical samples. Future research should also investigate the effects of priming in other groups, including men and women of different ethnic backgrounds, as well as individuals of different ages, given that they may be exposed to different types of body-ideal messaging in their environment or may respond to such messaging differently. Second, approximately 50% of the women recruited for the lab visit participated. Women who chose to participate in the lab visit may have differed from women who declined on dimensions that we did not measure, such as socioeconomic status, personality traits, or social engagement (Porter & Whitcomb, 2005). Although we know that women who declined to participate did not differ in ethnicity, age, BMI, body satisfaction, or body appreciation (see the “Participants” section), we cannot rule out the possibility that they differed in other important ways, and these differences could have influenced the composition of the high and low body dissatisfaction groups.

Third, with respect to the priming procedure, because we obtained no visual analog measure of state body satisfaction between the video and the writing activities that were part of each priming task, it is unclear what the relative contribution to the priming effect each of these components had. The writing tasks alone, in which participants wrote about their feelings toward their body, may have produced a stronger priming effect than the procedures used in previous studies (e.g., viewing images of fashion models). On the other hand, because participants’ written statements were private, some participants could have chosen not to engage in the writing activity, in which case any priming effect would have been entirely due to the videos. Future priming studies should keep these distinctions in mind, and perhaps explore variations of our priming tasks, using different videos, writing prompts, or instructions, to delineate the contribution of each component’s effects. Nevertheless, it is clear that the priming tasks as a whole were effective. Our results strongly suggest that future studies investigating body dissatisfaction priming should use similarly intensive priming procedures rather than thin model exposure alone.

The finding that the body satisfaction priming task did not reduce attentional biases in women with high levels of body dissatisfaction has implications for programs designed to reduce body dissatisfaction and prevent eating disorders in young women. Our results suggest that it may be more effective for programs to incorporate content that reduces the salience of body appearance in women’s lives and promotes body acceptance, while reducing inclinations to focus attention on the body. Priming body satisfaction appears to be more difficult than priming body dissatisfaction and may therefore require more elaborate procedures which integrate such strategies. Priming body satisfaction may be more difficult because of a general human propensity to devote more attention and react more strongly to negative information than to positive information (“bad is stronger than good”; Baumeister, Bratslavsky, Finkenauer, & Vohs, 2001). As a consequence, women may generally be more receptive to negative body-related messages than positive ones (especially women with elevated body dissatisfaction). If this interpretation is correct, then it would support the use of interventions that incorporate indirect approaches to change body image attitudes and attention.

## Conclusions

This study examined attention to body images before and after women experienced either a body satisfaction or body dissatisfaction priming task. As predicted, the body dissatisfaction priming task increased attention to thin and fat model images in women with high body dissatisfaction. Contrary to predictions, the body dissatisfaction priming task had the same effect for women with low body dissatisfaction, which suggests that these women are not necessarily immune from the effects of negative body image messages. The body satisfaction priming task had no effect on women’s attention to thin and fat model images, regardless of their degree of body dissatisfaction, which has implications for body image interventions. These findings support the continued investigation of attentional biases related to body dissatisfaction and their malleability. Additional research is necessary to devise body image interventions that can counteract attentional biases and reduce negative outcomes in women with high levels of body dissatisfaction.

## Notes

1. Research suggests that high levels of body appreciation may protect against body image threats, even among women who are vulnerable to thin ideal internalization and social comparison (e.g., Halliwell, 2013). We explored the potential role of body appreciation (as measured by the Body Appreciation Scale [BAS-2]; Tylka & Wood-Baraclow, 2015) in moderating the effect of body dissatisfaction priming on state body satisfaction using hierarchical multiple regression. In the analysis, the dependent variable was the change in state body satisfaction ratings following the body dissatisfaction priming (pre-prime vs. post-prime), with larger values reflecting a greater impact of the priming on participants’ state body satisfaction. For the first step, the two predictor variables were Body Dissatisfaction Group (women with low body dissatisfaction, women with high body dissatisfaction) and BAS-2 scores; the interaction term was added during the second step to assess for moderation. The interaction term was significant, *t*(50) = 2.53, *p* = .015. Follow-up analyses showed that for women with low body dissatisfaction, higher scores on the BAS-2 were associated with smaller changes in state body satisfaction ratings following the body dissatisfaction priming, *r*(20) = −.478, *p* = .025. That is, women with low body dissatisfaction and high body appreciation were less affected by the body dissatisfaction priming. In contrast, for women with high body dissatisfaction, there was no association between scores on the BAS-2 and post-prime changes in state body satisfaction ratings, *r*(30) = .162, *p* = .374. These results indicate that higher levels of body appreciation reduced the impact of the body dissatisfaction priming only for women with low body dissatisfaction. This outcome suggests that high levels of body appreciation in body-dissatisfied women may not always confer protection against body images threats.

2. To provide additional support for this conclusion, *t*-tests were used to confirm there was an effect of body dissatisfaction priming for women with high body dissatisfaction and for women with low body dissatisfaction. For women with high body dissatisfaction, total fixation times to thin model images increased after the body dissatisfaction priming (*M* = 2,548 ms vs. 2,982 ms), *t*(31) = 3.87, *p* < .01, and total fixation times to fat model images increased after the body dissatisfaction priming (*M* = 2,380 ms vs. 2,670 ms), *t*(31) = 2.10, *p* = .035. This was also true for women with low body dissatisfaction: total fixation times to thin model images increased after the body dissatisfaction priming (*M* = 2,188 ms vs. 2,484 ms), *t*(21) = 2.74, *p* = .012, as did total fixation times to fat model images (*M* = 2,026 ms vs. 2,312 ms), *t*(21) = 2.56, *p* = .018.
